# Association of Metabolic Score for Insulin Resistance With Resistant Hypertension and Hypertension in Obstructive Sleep Apnea

**DOI:** 10.1111/crj.70211

**Published:** 2026-07-09

**Authors:** Jinwei Xin, Rongxiu Xie, Wenxu Zhou, Jiaying Li, Chen Zhou, Taofeng Zhu

**Affiliations:** ^1^ Department of General Medicine Affiliated Yixing Hospital of Jiangsu University Yixing Jiangsu China; ^2^ Department of Respiratory and Critical Care Medicine Affiliated Yixing Hospital of Jiangsu University Yixing Jiangsu China

**Keywords:** hypertension, insulin resistance, metabolic score for insulin resistance, obstructive sleep apnea, polysomnography, resistant hypertension

## Abstract

**Introduction:**

Obstructive sleep apnea (OSA) is common in the presence of hypertension and refractory hypertension (RH); however, the pathophysiological interrelationship of such conditions is yet to be well explained with insulin resistance (IR) as a key mediator. The objective of the present investigation was (1) to evaluate the association of the metabolic score of insulin resistance (METS‐IR) with hypertension and RH in patients with OSA and (2) to understand whether this association varies according to the severity of OSA.

**Methods:**

It is a retrospective cohort, which included 680 adults with OSA diagnosis in 2020–2022. The participants were stratified into quartiles in accordance with their METS‐IR. Polysomnographic evaluation and metabolic profiling including body mass index (BMI), fasting blood glucose, triglycerides, and high‐density lipoprotein cholesterol (HDL‐C) were conducted. Multivariate logistic regression analyses were used to determine the relationship between METS‐IR and hypertension/RH, adjusting for sex, lifestyle, and variables related to OSA. Subgroup analyses were also done to compare associations between severe and non‐severe cases of OSA cohorts. Additionally, receiver operating characteristic (ROC) curve analysis was employed to compare the discriminative performance of METS‐IR, body mass index (BMI), and the triglyceride‐to‐high‐density lipoprotein cholesterol (TG/HDL) ratio for both hypertension and RH. Post hoc power analyses were conducted across all groups (the total cohort, the severe OSA subgroup, and the non‐severe OSA subgroup) to evaluate whether the analyses were adequately powered (> 80%) to detect the observed effect sizes.

**Results:**

Elevated METS‐IR levels were significantly associated with a higher prevalence of hypertension and RH. In patients with severe OSA, multivariate analysis revealed a robust, linear dose–response relationship between METS‐IR and the risks of both hypertension and RH (*p‐trend* < 0.01); conversely, in the non‐severe OSA group, these associations were attenuated and did not exhibit a significant linear trend after full adjustment. ROC analysis revealed that METS‐IR achieved the highest discriminative accuracy for both outcomes. For hypertension, the AUC of METS‐IR (0.745) was significantly higher than that of BMI (0.729) and TG/HDL (0.636) (all *p* < 0.05). Similarly, for RH, METS‐IR demonstrated superior discriminative ability (0.754) compared to BMI (0.746) and TG/HDL (0.610).

**Conclusions:**

METS‐IR is significantly associated with hypertension and RH in patients with severe OSA, independent of BMI, although this association is attenuated in patients with non‐severe OSA. These adequately powered results support the potential utility of METS‐IR as a simple metabolic marker to identify high‐risk phenotypes in clinical practice to manage severe OSA. Furthermore, METS‐IR is a more robust marker of hypertension and RH than BMI or TG/HDL alone, suggesting that the integration of adiposity and metabolic parameters provides superior risk stratification in OSA patients.

## Graphical abstract

This study identifies the metabolic score for insulin resistance (METS‐IR) as an independent predictor of hypertension and resistant hypertension in obstructive sleep apnea (OSA). Crucially, this dose–response relationship is robust in severe OSA but attenuated in non‐severe cases, highlighting METS‐IR as a practical biomarker for risk stratification in severe OSA phenotypes.

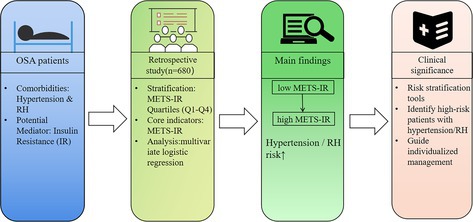

## 1 Background

Obstructive sleep apnea (OSA) is a widespread sleeping disorder whereby there is a recurrent collapse of the upper airway during sleep leading to apnea, hypoxemia, and sleep fragmentation/sympathetic activation [1]. OSA not only impairs sleep quality but is also strongly associated with a strong relation to the emergence of multiple metabolic and cardiovascular diseases [2]. One of these comorbidities is hypertension and especially resistant hypertension (RH) that has a greater prevalence in the population of OSA patients. Although the association between OSA and hypertension is fully established, the exact underlying different mechanisms are not fully understood [3].

Recently, insulin resistance (IR) has become an inseparable element of metabolic syndrome and a significant pathway in explaining the interplay between OSA and hypertension [4]. Proper evaluation of insulin resistance is critical to the study of diabetes, metabolic syndrome, and cardiovascular diseases [5]. Conventional strategies of assessing IR are primarily based on fasting plasma glucose (FPG), insulin levels, and correlative indices, but these markers fail to fully reflect the multifaceted nature of metabolic perturbations [6]. A new insulin resistance marker, therefore, the metabolic score of insulin resistance (METS‐IR), is created. This score is a combination of various metabolic indices such as FPG, triglycerides (TG), high‐density lipoprotein cholesterol (HDL‐C), and body mass index (BMI) to give a more multifaceted display of the metabolic condition [7]. The previously existing literature has already proved that METS‐IR can accurately assess the degree of insulin resistance and is strongly associated with the predictability of cardiovascular and metabolic illnesses occurrence [8].

Metabolic disorders frequently occur in patients with OSA, insulin resistance being one of the strongest ones [2]. This can help promotion and progression of hypertension by means of increased sympathetic nervous activity, endothelial dysfunction, and inflammatory reactions, thus aggravating the condition [9]. To further clarify the importance of insulin resistance in OSA patients being comorbid with hypertension and RH, the current study will address METS‐IR as an indicator of insulin resistance in the mentioned cohort and will also investigate its pathophysiology. Through the help of stringent statistical procedures, this study aims to investigate the association of METS‐IR in this cohort, exploring its potential as a metabolic indicator to aid clinical risk stratification.

## 2 Methods

### 2.1 Design and Population of the Studies

The current retrospective analysis filtered sequential adult people (aged 18 years or older) with the diagnosis of OSA between January 2020 and December 2022 at the Affiliated Yixing Hospital of Jiangsu University. OSA was defined as an apnea–hypopnea index (AHI) ≥ 5 events/h polysomnography (PSG) with the common symptoms, according to the guidelines of the American Academy of Sleep Medicine (AASM) [10].

Patients with hypertension were included according to the ACC/AHA guidelines, defined as an office blood pressure ≥ 130/80 mmHg or current use of antihypertensive medication [11]. A subgroup of patients with RH was identified based on the 2020 International Society of Hypertension criteria, characterized by uncontrolled blood pressure despite the use of three or more antihypertensive agents, including a diuretic [12].

**FIGURE 1:**
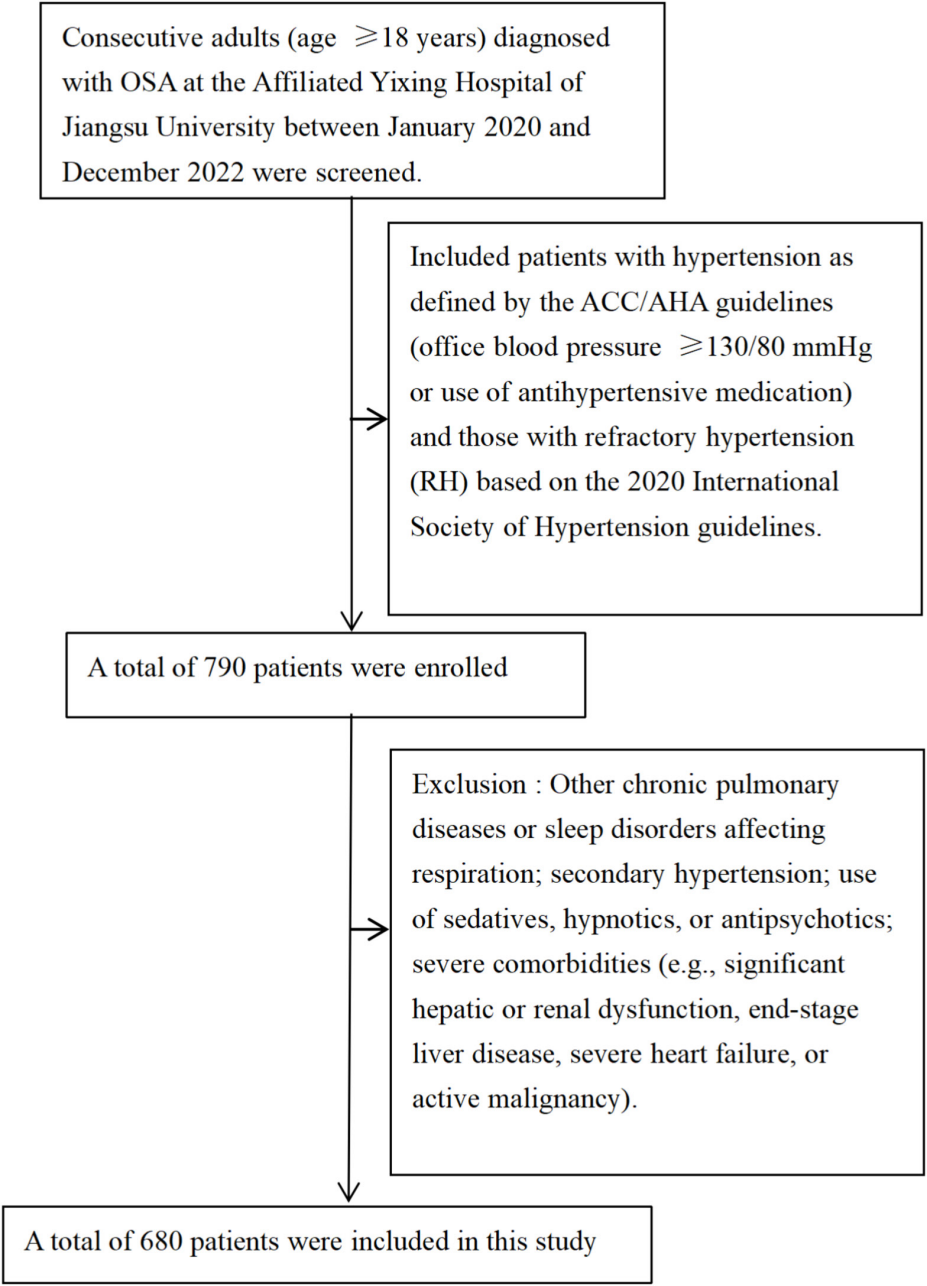
The patient screening process.

Patients were excluded from this study if they met any of the following criteria: (1) presence of co‐existing chronic respiratory diseases (e.g., COPD and asthma) or other sleep disorders that could interfere with respiratory monitoring; (2) diagnosis of secondary hypertension; (3) current use of sedatives, hypnotics, or antipsychotics, which may alter sleep architecture or respiratory drive; or (4) severe comorbidities, including advanced renal or hepatic insufficiency, heart failure (NYHA Class III or IV), or active malignancy. Only patients with complete clinical data were included (Figure 1).

Finally, a total of 680 eligible patients were enrolled. According to the quartiles of the METS‐IR, participants were categorized into four groups: M1, M2, M3, and M4, with 170 patients in each group.

### 2.2 Data Collection and Measurements

All the participants were systemically entered with demographic features, lifestyle habits, and health histories that included their age, sex, BMI, smoking, drinking habits, and the cerebral infarction, diabetes mellitus, and CVD (cardiovascular disease).

To calculate total cholesterol (TC), TG, HDL‐C, low‐density lipoprotein cholesterol (LDL‐C), and FPG values, venous blood samples were collected in the morning after no less than 8 h of fasting. A Siemens ADVIA 1200 automated biochemical analyzer (Siemens Healthcare Diagnostics, Germany) was automated to measure all the biochemical parameters.

### 2.3 METS‐IR

METS‐IR was estimated by using the equation initially proposed: ln[2 × FPG + TG × BMI/ln[HDL‐C] [13]. Because our laboratory results of FPG, TG, and HDL‐C are in mmol/L, the results were transformed to mg/dL by using standardized conversion factors (FPG and TG: ×18.018; HDL‐C: ×38.67). This weighted index provides an overall measure of metabolic condition as a combination of insulin resistance, dyslipidemia, and adiposity pathways.

### 2.4 Sleep Study

Overnight PSG was conducted on all the participants in the Affiliated Yixing Hospital of Jiangsu University under the SOMNOlab 2 system (Weinmann Emergency, Germany). Parameters such as AHI, total sleep time (TST), percentage of total time with oxygen saturation level < 90% (T90), mean pulse oxygen saturation (MSaO_2_), lowest pulse oxygen saturation (LSaO_2_), and the oxygen desaturation index (ODI) were calculated by analyzing PSG recordings. The patients were requested not to take any form of sedative drugs before the start of the monitoring, which started at 21:00 on the previous day and was maintained until 07:00 the next morning. The subjective Epworth Sleepiness Scale (ESS) score questionnaire is used to fully characterize sleep disturbances as it was supplemented with objective PSG data [14].

### 2.5 Statistical Analysis

Coding and subsequent management of the data were done with Microsoft Excel 2021, with statistical calculations being done using SPSS22.0. Histograms and Q‐Q plots were used to assess the normality of continuous variables. Normally distributed variables have been summarized as mean ± the standard deviation (mean ± SD); the comparison of multiple groups was done with one‐way analysis of variance (ANOVA). The values of the non‐normally distributed variables were presented in the form of medians and interquartile ranges of Median (Q1 and Q3), and the Kruskal–Wallis test was used in group comparisons. The categorical variables were in the form of counts (percentages) where the differences between intergroup were evaluated through the Pearson chi‐square (*χ* ^*2*^) test.

The METS‐IR quartiles were used to classify the participants into four groups (M1, M2, M3, and M4). Multivariate logistic regression analysis was performed to determine the independent relationship between METS‐IR and the prevalence of hypertension or RH in OSA patients and adjusting possible confounders. In this model, the METS‐IR quartiles were considered an ordinal variable in order to determine changes in risk. Moreover, the study cohort was also stratified into severe versus non‐severe OSA subgroups (severe OSA = AHI ≥ 30) in order to compare the strength of the association of METS‐IR in relation to risk of hypertension/RH at different levels of disease severity. To evaluate the incremental value of the composite METS‐IR over BMI, ROC curves were separately constructed for two clinical outcomes: hypertension and RH. The area under the curve (AUC) was calculated for both METS‐IR, BMI, and the triglyceride‐to‐high‐density lipoprotein cholesterol (TG/HDL) ratio to assess their ability to identify hypertension and RH. All analyses were performed using GraphPad Prism 10.1.2. Beyond that, post hoc power analyses were performed using G*Power software (Version 3.1.9.7) to evaluate the statistical reliability of our findings across all study populations. For the quartile‐based logistic regression models, the achieved power (1‐β) was calculated based on the observed effect sizes (odds ratios) and the specific sample sizes involved in the comparison between the highest (M4) and lowest (M1) quartiles. A power value > 0.80 was pre‐specified as the threshold for an adequately powered analysis to detect the observed associations. This post hoc evaluation was applied to the total cohort, the severe OSA subgroup, and the non‐severe OSA subgroup to interpret both significant and nonsignificant results.

Two‐tailed *p*‐value below 0.05 was believed to be significant.

## 3 Results

### 3.1 Baseline Characteristics of OSA Patients Across METS‐IR Groups

A total of 680 participants were included, comprising 81.3% males and 18.7% females. Baseline analysis showed significant differences in sociodemographic and lifestyle factors across groups, with a higher proportion of males (*p* = 0.024) and increasing rates of smoking and alcohol consumption with higher METS‐IR quartiles (all *p* < 0.01). The prevalence of hypertension, RH, cerebral infarction, diabetes, and CVD was markedly higher in high METS‐IR groups (all *p* < 0.001), reaching 88.8%, 37.6%, 62.9%, 41.2%, and 50.0%, respectively, in the M4 group. BMI, FPG, TC, and TG levels rose, while HDL‐C declined with increasing METS‐IR (all *p* < 0.001); LDL‐C showed no significant difference (*p* = 0.337). Sleep parameters, including ESS score, AHI, ODI, and T90, increased with METS‐IR, whereas LSaO2 and MSaO2 decreased (all *p* < 0.001). No significant difference was observed in TST (*p* = 0.316). Detailed data are shown in Table 1.

**TABLE 1:** Baseline data of different METS‐IR groups.

	Overall	METS‐IR					
		M1	M2	M3	M4	Statistic	*p*
Age (years), mean±SD	53.5 ± 13.7	56.3 ± 14.2	53.7 ± 12.4	54.7 ± 13.4	49.2 ± 13.7	8.711^c^	< 0.001
Male, *n* (%)	553 (81.3)	127 (74.7)	149 (87.6)	139 (81.8)	138 (81.2)	9.402^a^	0.024
Hypertension, *n* (%)	518 (76.2)	88 (51.8)	133 (78.2)	146 (85.9)	151 (88.8)	80.029^a^	< 0.001
RH, *n* (%)	129 (19.0)	9 (5.3)	20 (11.8)	36 (21.2)	64 (37.6)	65.542^a^	< 0.001
Smoking, *n* (%)	249 (36.6)	46 (27.1)	60 (35.3)	64 (37.6)	79 (46.5)	14.009^a^	0.003
Alcohol consumption, *n* (%)	86 (12.6)	13 (7.6)	16 (9.4)	18 (10.6)	39 (22.9)	22.416^a^	< 0.001
Cerebral infarction, *n* (%)	298 (43.8)	39 (22.9)	62 (36.5)	90 (52.9)	107 (62.9)	64.824^a^	< 0.001
Diabetes, *n* (%)	140 (20.6)	9 (5.3)	16 (9.4)	45 (26.5)	70 (41.2)	84.982^a^	< 0.001
CVD, *n* (%)	189 (27.8)	18 (10.6)	32 (18.8)	54 (31.8)	85 (50.0)	74.999^a^	< 0.001
BMI (kg/m^2^), mean±SD	27.5 ± 4.0	23.3 ± 2.2	26.1 ± 1.6	28.4 ± 1.8	32.2 ± 3.4	434.979^c^	< 0.001
METS‐IR, mean±SD	43.7 ± 8.2	34.3 ± 3.4	40.8 ± 1.5	45.7 ± 1.5	54.2 ± 6.2	883.958^c^	< 0.001
AHI (events/h), median (Q1, Q3)	25.2 (13.7, 42.8)	23.2 (13.0, 34.7)	24.7 (14.6, 37.8)	25.7 (13.0, 46.4)	30.4 (14.5, 47.6)	12.557^b^	0.006
ESS, median (Q1, Q3)	10.0 (8.0, 13.0)	9.0 (7.0, 12.0)	10.0 (8.0, 12.0)	11.0 (8.0, 13.1)	11.5 (9.0, 14.0)	30.641^b^	< 0.001
TST (h), median (Q1, Q3)	8.0 (6.6, 8.7)	8.0 (6.7, 8.7)	7.9 (6.5, 8.7)	8.0 (6.6, 8.8)	7.8 (6.4, 8.5)	3.538^b^	0.316
T90, median (Q1, Q3)	2.1 (0.4, 9.5)	0.8 (0.2, 3.3)	1.6 (0.2, 6.4)	3.4 (0.5, 15.3)	5.8 (1.3, 20.2)	71.522^b^	< 0.001
LSaO_2_ (%), median (Q1, Q3)	78.0 (68.0, 84.8)	82.0 (74.0, 86.0)	81.0 (71.8, 86.3)	77.0 (67.8, 83.3)	73.0 (62.0, 83.0)	44.854^b^	< 0.001
MSaO_2_ (%), median (Q1, Q3)	95.3 (94.0, 96.2)	96.0 (95.0, 97.0)	96.0 (94.9, 96.8)	95.1 (93.7, 96.0)	94.4 (92.0, 95.6)	86.468^b^	< 0.001
ODI (events/h), median (Q1, Q3)	14.3 (5.7, 30.6)	7.6 (3.3, 17.3)	12.0 (4.5, 23.8)	20.9 (7.9, 34.9)	21.7 (11.3, 45.2)	83.398^b^	< 0.001
TC (mmol/L), mean±SD	4.4 ± 1.0	4.2 ± 1.0	4.2 ± 0.9	4.3 ± 0.9	4.7 ± 1.2	8.067^c^	< 0.001
TG (mmol/L), median (Q1, Q3)	1.7 (1.2, 2.5)	1.2 (0.9, 1.6)	1.7 (1.2, 2.3)	1.7 (1.2, 2.4)	2.4 (1.5, 3.6)	130.553^b^	< 0.001
HDL‐C (mmol/L), mean±SD	1.1 ± 0.2	1.3 ± 0.3	1.1 ± 0.2	1.0 ± 0.2	1.0 ± 0.2	52.803^c^	< 0.001
LDL‐C (mmol/L), mean±SD	2.6 ± 0.8	2.5 ± 0.8	2.6 ± 0.7	2.6 ± 0.7	2.6 ± 0.8	1.129^c^	0.337
FPG (mmol/L), median (Q1, Q3)	5.4 (4.8, 6.3)	5.1 (4.5, 5.6)	5.4 (4.8, 6.2)	5.5 (4.9, 6.3)	5.8 (5.1, 7.1)	53.385^b^	< 0.001

Abbreviations: AHI = apnea–hypopnea index, BMI = body mass index, CVD = cardiovascular disease, ESS = Epworth Sleepiness Scale, FPG = fasting plasma glucose, HDL‐C = high‐density lipoprotein cholesterol, LDL‐C = low‐density lipoprotein cholesterol, LSaO2 = lowest pulse oxygen saturation, METS‐IR = metabolic score of insulin resistance, MSaO2 = mean pulse oxygen saturation, ODI = oxygen desaturation index, RH = resistant hypertension, TC = total cholesterol, TG = triglyceride, TST = total sleep time, T90 = percentage of total time with oxygen saturation level < 90%.

^a^ χ^2^ test.

^b^ Kruskal–Wallis test.

^c^ ANOVA.

### 3.2 Association Between METS‐IR and Hypertension in OSA Patients

Multivariable logistic regression analysis was performed with hypertension as the dependent variable and METS‐IR as the independent variable, incorporating the statistically significant covariates identified in the univariate analysis. Given the intrinsic correlation between METS‐IR and its components (FPG, TG, BMI, and HDL‐C), these variables were excluded from the regression models to avoid multicollinearity. A multi‐model analytical approach was adopted: Model 1: unadjusted (crude model); Model 2: adjusted for age, sex, smoking, alcohol consumption, cerebral infarction, diabetes, and CVD; and Model 3: further adjusted for sleep‐related parameters, including AHI, ESS score, T90, LSaO2, MSaO2, ODI, and TC, based on Model 2.

Using the lowest METS‐IR quartile (M1 group) as the reference, the regression analysis demonstrated that higher METS‐IR levels were significantly associated with an increased risk of hypertension. In the unadjusted Model 1, the ORs for hypertension in the M2, M3, and M4 groups were 3.350 (95% CI: 2.088–5.372), 5.669 (95% CI: 3.349–9.594), and 7.406 (95% CI: 4.213–13.017), respectively (all *p* < 0.001). These significant associations persisted even after adjusting for potential confounders in both Model 2 and Model 3 (all *p* < 0.001).

A trend test revealed a significant linear dose–response relationship between increasing METS‐IR levels and the risk of hypertension (*p‐trend* < 0.001), indicating that a high METS‐IR is an independent associated factor for hypertension in patients with OSA. Detailed results are presented in Table 2.

**TABLE 2:** Multivariate logistic regression analysis of METS‐IR level on OSA complicated with hypertension.

METS‐IR/hypertension	Model 1		Model 2		Model 3	
	OR (95% CI)	*p*	OR (95% CI)	*p*	OR (95% CI)	*p*
M1	—	—	—	—	—	—
M2	3.350 (2.088, 5.372)	< 0.001	3.542 (2.159, 5.811)	< 0.001	3.687 (2.216, 6.134)	< 0.001
M3	5.669 (3.349, 9.594)	< 0.001	6.091 (3.425, 10.832)	< 0.001	5.915 (3.215, 10.881)	< 0.001
M4	7.406 (4.213, 13.017)	< 0.001	7.519 (3.824, 14.784)	< 0.001	6.621 (3.193, 13.733)	< 0.001
*p‐trend*		< 0.001		< 0.001		< 0.001

*Note:* Model 1: unadjusted (crude model); Model 2: adjusted for age, sex, smoking, alcohol consumption, cerebral infarction, diabetes, and CVD; and Model 3: further adjusted for sleep‐related parameters, including AHI, ESS score, T90, LSaO2, MsaO2, ODI, and TC, based on Model 2.

### 3.3 Association Between METS‐IR and Resistant Hypertension in OSA Patients

Similarly, a multivariable logistic regression analysis was performed with RH as the dependent variable, incorporating the same statistically significant covariates. The results are as follows:

In the crude model (Model 1), ORs for RH in the M2, M3, and M4 groups were 2.385 (95% CI: 1.053–5.402, *p* = 0.037), 4.806 (95% CI: 2.235–10.334, *p* < 0.001), and 10.801 (95% CI: 5.155–22.628, *p* < 0.001), respectively, indicating a significantly increased risk of RH with elevated METS‐IR levels. These associations remained significant in Model 2 after adjusting for non‐sleep‐related confounders, with ORs of 2.316 (95% CI: 1.007–5.325, *p* = 0.048) for M2, 4.519 (95% CI: 2.029–10.067, *p* < 0.001) for M3, and 9.439 (95% CI: 4.115–21.651, *p* < 0.001) for M4. However, in the fully adjusted Model 3 (which included sleep parameters), the risk for the M2 group was no longer statistically significant 2.253 (95% CI: 0.939–5.407, *p* = 0.069), while the risks for the M3 and M4 groups remained significantly elevated, with ORs of 3.034 (95% CI: 1.276–7.215, *p* = 0.012) and 5.142 (95% CI: 2.085–12.680, *p* < 0.001), respectively.

A significant linear dose–response relationship was observed between increasing METS‐IR quartiles and the risk of RH (*p‐trend* < 0.001), suggesting that a high METS‐IR is an independent associated factor for RH. Detailed results are presented in Table 3.

**TABLE 3:** Multivariate logistic regression analysis of METS‐IR level on OSA with RH.

METS‐IR/RH	Model 1		Model 2		Model 3	
	OR (95% CI)	*p*	OR (95% CI)	*p*	OR (95% CI)	*p*
M1	—	—	—	—	—	—
M2	2.385 (1.053, 5.402)	0.037	2.316 (1.007, 5.325)	0.048	2.253 (0.939, 5.407)	0.069
M3	4.806 (2.235, 10.334)	< 0.001	4.519 (2.029, 10.067)	< 0.001	3.034 (1.276, 7.215)	0.012
M4	10.801 (5.155, 22.628)	< 0.001	9.439 (4.115, 21.651)	< 0.001	5.142 (2.085, 12.680)	< 0.001
*p‐trend*		< 0.001		< 0.001		< 0.001

*Note:* Model 1: unadjusted (crude model); Model 2: adjusted for age, sex, smoking, alcohol consumption, cerebral infarction, diabetes, and CVD; and Model 3: further adjusted for sleep‐related parameters, including AHI, ESS score, T90, LSaO2, MSaO2, ODI, and TC, based on Model 2.

Abbreviation: RH = resistant hypertension.

### 3.4 Association of METS‐IR on Hypertension and RH in Severe OSA

This analysis included 288 patients with severe OSA, distributed across METS‐IR quartiles as follows: 59 (20.49%) in M1, 64 (22.22%) in M2, 79 (27.43%) in M3, and 86 (29.86%) in M4. Significant differences (*p* < 0.05) were observed among the four groups in age, BMI, prevalence of hypertension, RH, cerebral infarction, diabetes, CVD, smoking status, alcohol consumption, ESS score, AHI, T90, LSaO2, MSaO2, ODI, TC, TG, HDL‐C, FPG, and METS‐IR itself. No significant differences were found in sex, TST, or LDL‐C levels (*p* > 0.05). For detailed data, see Table 4.

**TABLE 4:** Baseline data of severe OSA group.

	Overall	METS‐IR					
		M1 *N* = 59	M2 *N* = 64	M3 *N* = 79	M4 *N* = 86	Statistic	*p*
Age (years), mean±SD	52.9 ± 12.7	55.1 ± 13.4	52.3 ± 12.0	55.0 ± 13.0	49.9 ± 12.0	3.060^c^	0.029
Male, *n* (%)	250 (86.8)	53 (89.8)	56 (87.5)	67 (84.8)	74 (86.0)	0.816^a^	0.846
Hypertension, *n* (%)	190 (66.0)	5 (8.5)	40 (62.5)	67 (84.8)	78 (90.7)	123.140^a^	< 0.001
RH, *n* (%)	79 (27.4)	3 (5.1)	9 (14.1)	25 (31.6)	42 (48.8)	41.048^a^	< 0.001
Smoking, *n* (%)	119 (41.3)	16 (27.1)	23 (35.9)	32 (40.5)	48 (55.8)	13.145^a^	0.004
Alcohol consumption, *n* (%)	40 (13.9)	5 (8.5)	7 (10.9)	8 (10.1)	20 (23.3)	9.156^a^	0.027
Cerebral infarction, *n* (%)	143 (49.7)	15 (25.4)	27 (42.2)	44 (55.7)	57 (66.3)	25.946^a^	< 0.001
Diabetes, *n* (%)	68 (23.6)	4 (6.8)	8 (12.5)	22 (27.8)	34 (39.5)	26.525^a^	< 0.001
CVD, *n* (%)	93 (32.3)	11 (18.6)	16 (25.0)	25 (31.6)	41 (47.7)	15.905^a^	0.001
BMI (kg/m^2^), mean±SD	28.0 ± 4.2	23.1 ± 2.0	26.0 ± 1.5	28.6 ± 1.7	32.2 ± 3.7	197.536^c^	< 0.001
METS‐IR, mean±SD	44.9 ± 8.7	34.3 ± 3.2	40.7 ± 1.6	45.7 ± 1.6	54.8 ± 7.2	336.967^c^	< 0.001
AHI (events/h), median (Q1, Q3)	44.8 (37.0, 53.6)	39.6 (34.3, 45.7)	43.8 (35.9, 50.0)	46.6 (38.9, 56.8)	47.5 (38.8, 61.8)	24.412^b^	< 0.001
ESS, median (Q1, Q3)	13.0 (11.0, 14.0)	12.0 (10.0, 14.0)	12.5 (11.0, 14.0)	13.0 (12.0, 15.0)	14.0 (12.0, 15.0)	14.587^b^	0.002
TST(h), median (Q1, Q3)	7.6 (6.0, 8.5)	7.5 (5.8, 8.6)	7.7 (6.0, 8.6)	7.8 (6.2, 8.6)	7.5 (6.1, 8.5)	0.786^b^	0.853
T90, median (Q1, Q3)	6.3 (1.27, 23.0)	1.8 (0.4, 7.3)	3.6 (0.7, 15.3)	9.3 (2.3, 29.4)	11.5 (2.4, 34.8)	33.353^b^	< 0.001
LSaO_2_ (%), median (Q1, Q3)	74.0 (63.0, 82.0)	80.0 (70.0, 85.0)	76.0 (64.0, 85.0)	73.0 (64.0, 80.0)	66.5 (60.0, 78.0)	22.110^b^	< 0.001
MSaO_2_ (%), median (Q1, Q3)	94.8 (92.6, 96.0)	95.8 (94.0, 97.0)	95.1 (94.0, 96.0)	94.0 (92.2, 95.3)	93.1 (90.4, 95.0)	45.897^b^	< 0.001
ODI (events/h), median (Q1, Q3)	28.9 (12.6, 47.1)	17.2 (5.0, 29.1)	22.4 (8.2, 39.2)	33.4 (23.3, 49.7)	41.4 (18.2, 56.3)	40.517^b^	< 0.001
TC (mmol/L), mean±SD	4.4 ± 1.0	4.0 ± 0.9	4.4 ± 0.8	4.4 ± 1.0	4.7 ± 1.2	4.678^c^	0.003
TG (mmol/L), median (Q1, Q3)	1.6 (1.1, 2.4)	1.2 (1.0, 1.6)	1.5 (1.2, 2.2)	1.6 (1.2, 2.3)	2.3 (1.5, 4.2)	54.476^b^	< 0.001
HDL‐C (mmol/L), mean±SD	1.1 ± 0.2	1.2 ± 0.3	1.1 ± 0.2	1.0 ± 0.2	1.0 ± 0.2	18.155^c^	< 0.001
LDL‐C (mmol/L), mean±SD	2.6 ± 0.7	2.5 ± 0.7	2.7 ± 0.7	2.7 ± 0.7	2.5 ± 0.7	1.785^c^	0.150
FPG (mmol/L), median (Q1, Q3)	5.4 (4.9, 6.4)	5.2 (4.9, 5.6)	5.2 (4.7, 6.0)	5.5 (4.9, 6.2)	6.1 (5.2, 7.4)	26.180^b^	< 0.001

Abbreviations: AHI = apnea–hypopnea index, BMI = body mass index, CVD = cardiovascular disease, ESS = Epworth Sleepiness Scale, FPG = fasting plasma glucose, HDL‐C = high‐density lipoprotein cholesterol, LDL‐C = low‐density lipoprotein cholesterol, LSaO2 = lowest pulse oxygen saturation, METS‐IR = metabolic score of insulin resistance, MSaO2 = mean pulse oxygen saturation, ODI = oxygen desaturation index, RH = resistant hypertension, TC = total cholesterol, TG = triglyceride, TST = total sleep time, T90 = percentage of total time with oxygen saturation level < 90%.

^a^ χ^2^ test.

^b^ Kruskal–Wallis test.

^c^ ANOVA.

Multivariable logistic regression analyses were performed, incorporating clinically relevant variables from the baseline characteristics. Model 1 provided unadjusted estimates. Model 2 was adjusted for age, smoking, alcohol consumption, cerebral infarction, diabetes, and CVD. Model 3 was further adjusted for sleep‐respiratory parameters and lipid profiles.

For Hypertension: In Model 1, the risks for hypertension in the M2, M3, and M4 groups were 18.000 (*95%CI*:6.320–51.267, *p* < 0.001), 60.300 (*95%CI*:20.009–181.718, *p* < 0.001), and 105.300 (*95%CI*:32.682–339.270, *p* < 0.001), respectively, demonstrating a markedly increased risk with higher METS‐IR levels. These associations remained significant in Model 2 (M2: *OR* = 19.121, *95%CI*:6.471–56.502, *p* < 0.001; M3: *OR* = 61.405, 95%CI:19.350–194.860, *p* < 0.001; M4: *OR* = 102.822, *95%CI*:28.586–369.840, *p* < 0.001) and Model 3 (M2: *OR* = 19.468, *95%CI*:6.231–60.826, *p* < 0.001; M3: *OR* = 49.547, *95%CI*:14.362–170.928, *p* < 0.001; M4: *OR* = 73.296, *95%CI*:18.906–284.152, *p* < 0.001), indicating a robust positive correlation.

For RH: In Model 1, the risks for RH were significantly elevated in the M3 (*OR* = 8.642, *95%CI*:2.465–30.301, *p* = 0.001) and M4 (*OR* = 17.818, *95%CI*:5.177–61.331, *p* < 0.001) groups compared to the M1 reference, while the risk in the M2 group was not significant (*OR* = 3.055, *95%CI*:0.785–11.884, *p* = 0.107). This pattern persisted in Model 2, with significant risks for M3 (*OR* = 7.680, *95%CI*:2.075–28.417, *p* = 0.002) and M4 (*OR* = 16.566, *95%CI*:4.325–63.449, *p* < 0.001), but not for M2 (*OR* = 2.640,*95%CI*:0.653–10.679, *p* = 0.173). In Model 3, the risk for RH remained significant for the M4 group (*OR* = 9.452, *95%CI*:1.971–45.335, *p* = 0.005) and was marginally non‐significant for M3 (*OR* = 4.446, *95%CI*:0.975–20.278, *p* = 0.054), while the M2 group risk remained non‐significant (*OR* = 3.079, *95%CI*:0.644–14.713, *p* = 0.159).

Trend tests confirmed a significant linear dose–response relationship between increasing METS‐IR levels and the risk of both hypertension and RH (*P‐trend* < 0.01), further substantiating METS‐IR as an independent associated factor in patients with severe OSA. Detailed results are presented in Table 5.

**TABLE 5:** Multivariate logistic regression analysis of METS‐IR level on OSA complicated with hypertension/RH in severe OSA group.

METS‐IR/hypertension, RH	Hypertension						RH					
	Model 1		Model 2		Model 3		Model 1		Model 2		Model 3	
	OR (95% CI)	*p*	OR (95% CI)	*p*	OR (95% CI)	*p*	OR (95% CI)	*p*	OR (95% CI)	*p*	OR (95% CI)	*p*
M1	—	—	—	—	—	—	—	—	—	—	—	—
M2	18.000 (6.320, 51.267)	< 0.001	19.121 (6.471, 56.502)	< 0.001	19.468 (6.231, 60.826)	< 0.001	3.055 (0.785, 11.884)	0.107	2.640 (0.653, 10.679)	0.173	3.079 (0.644, 14.713)	0.159
M3	60.300 (20.0091, 81.718)	< 0.001	61.405 (19.3501, 94.860)	< 0.001	49.547 (14.3621, 70.928)	< 0.001	8.642 (2.465, 30.301)	0.001	7.680 (2.075, 28.417)	0.002	4.446 (0.975, 20.278)	0.054
M4	105.300 (32.682, 339.270)	< 0.001	102.822 (28.586, 369.840)	< 0.001	73.296 (18.9062, 84.152)	< 0.001	17.818 (5.177, 61.331)	< 0.001	16.566 (4.325, 63.449)	< 0.001	9.452 (1.971, 45.335)	0.005
*p‐trend*		< 0.001		< 0.001		< 0.001		< 0.001		< 0.001		0.002

*Note:* Model 1 provided unadjusted estimates; Model 2 was adjusted for age, smoking, alcohol consumption, cerebral infarction, diabetes, and CVD; and Model 3 was further adjusted for sleep‐related parameters, including AHI, ESS score, T90, LSaO2, MSaO2, ODI, and TC, based on Model 2.

Abbreviation: RH = resistant hypertension.

### 3.5 Association of METS‐IR on Hypertension and RH in Non‐Severe OSA

This analysis included 392 patients with non‐severe OSA, distributed across METS‐IR quartiles as follows: 111 (28.32%) in M1, 106 (27.04%) in M2, 91 (23.21%) in M3, and 84 (21.43%) in M4. Significant differences (*p* < 0.05) were observed among the quartiles in age, sex, BMI, and the prevalence of hypertension, RH, cerebral infarction, diabetes, CVD, as well as in alcohol consumption, T90, LSaO2, MSaO2, ODI, TC, TG, HDL‐C, FPG, and METS‐IR itself. No significant differences were found in smoking status, AHI, ESS score, TST, or LDL‐C levels (*p* > 0.05). For detailed data, see Table 6.

**TABLE 6:** Baseline data of non‐severe OSA group.

	Overall	METS‐IR					
		M1 *N* = 111	M2 *N* = 106	M3 *N* = 91	M4 *N* = 84	Statistic	*p*
Age (years), mean±SD	54.0 ± 14.3	57.0 ± 14.6	54.6 ± 12.6	54.5 ± 13.9	48.6 ± 15.2	5.815^c^	0.001
Male, *n* (%)	303 (77.3)	74 (66.7)	93 (87.7)	72 (79.1)	64 (76.2)	13.961^a^	0.003
Hypertension, *n* (%)	328 (83.7)	83 (74.8)	93 (87.7)	79 (86.8)	73 (86.9)	9.013^a^	0.029
RH, *n* (%)	50 (12.8)	6 (5.4)	11 (10.4)	11 (12.1)	22 (26.2)	19.589^a^	< 0.001
Smoking, *n* (%)	130 (33.2)	30 (27.0)	37 (34.9)	32 (35.2)	31 (36.9)	2.726^a^	0.436
Alcohol consumption, *n* (%)	46 (11.7)	8 (7.2)	9 (8.5)	10 (11.0)	19 (22.6)	12.930^a^	0.005
Cerebral infarction, *n* (%)	155 (39.5)	24 (21.6)	35 (33.0)	46 (50.6)	50 (59.5)	35.439^a^	< 0.001
Diabetes, *n* (%)	72 (18.4)	5 (4.5)	8 (7.5)	23 (25.3)	36 (42.9)	59.000^a^	< 0.001
CVD, *n* (%)	96 (24.5)	7 (6.3)	16 (15.1)	29 (31.9)	44 (52.4)	62.922^a^	< 0.001
BMI (kg/m^2^), mean±SD	27.1 ± 3.9	23.4 ± 2.2	26.1 ± 1.7	28.3 ± 2.0	32.1 ± 3.2	238.191^c^	< 0.001
METS‐IR, mean±SD	42.8 ± 7.7	34.2 ± 3.6	40.9 ± 1.4	45.7 ± 1.5	53.6 ± 4.9	618.661^c^	< 0.001
AHI (events/h), median (Q1, Q3)	15.3 (10.2, 22.3)	15.4 (10.4, 22.9)	15.9 (11.7, 22.7)	14.2 (8.7, 20.5)	14.2 (9.7, 22.9)	5.430^b^	0.143
ESS, median (Q1, Q3)	8.0 (7.0, 10.0)	8.0 (6.0, 10.0)	8.0 (7.0, 10.0)	8.0 (6.0, 11.0)	9.0 (7.0, 11.0)	6.961^b^	0.073
TST(h), median (Q1, Q3)	8.0 (7.0, 8.8)	8.0 (7.1, 8.8)	8.0 (6.5, 8.8)	8.4 (7.0, 9.0)	8.0 (7.0, 8.5)	3.233^b^	0.357
T90, median (Q1, Q3)	1.1 (0.2, 4.3)	0.6 (0.0, 1.9)	0.9 (0.0, 3.3)	1.2 (0.3, 5.0)	3.1 (0.8, 8.7)	34.014^b^	< 0.001
LSaO_2_ (%), median (Q1, Q3)	81.0 (72.0, 86.0)	82.0 (75.0, 87.0)	82.0 (76.8, 88.0)	79.0 (73.0, 85.0)	78.0 (66.0, 84.0)	16.591^b^	< 0.001
MSaO_2_ (%), median (Q1, Q3)	96.0 (94.9, 96.9)	96.0 (95.0, 97.0)	96.0 (95.2, 97.0)	95.8 (94.5, 96.5)	95.0 (93.8, 96.0)	33.549^b^	< 0.001
ODI (events/h), median (Q1, Q3)	9.5 (3.8, 17.5)	5.8 (2.7, 11.8)	9.1 (3.4, 14.4)	10.4 (5.1, 23.3)	15.5 (7.6, 25.6)	37.227^b^	< 0.001
TC (mmol/L), mean±SD	4.3 ± 1.0	4.4 ± 1.0	4.1 ± 0.9	4.2 ± 0.9	4.7 ± 1.2	6.005^c^	0.001
TG (mmol/L), median (Q1, Q3)	1.7 (1.2, 2.4)	1.2 (0.9, 1.7)	1.8 (1.3, 2.4)	1.9 (1.4, 2.5)	2.4 (1.6, 3.3)	79.494^b^	< 0.001
HDL‐C (mmol/L), mean±SD	1.1 ± 0.3	1.3 ± 0.3	1.1 ± 0.2	1.0 ± 0.2	1.0 ± 0.2	32.725^c^	< 0.001
LDL‐C (mmol/L), mean±SD	2.5 ± 0.8	2.5 ± 0.8	2.5 ± 0.6	2.5 ± 0.7	2.7 ± 1.0	1.615^c^	0.186
FPG (mmol/L), median (Q1, Q3)	5.5 (4.7, 6.2)	4.9 (4.5, 5.6)	5.5 (4.8, 6.4)	5.7 (5.0, 6.3)	5.7 (4.9, 6.5)	34.845^b^	< 0.001

Abbreviations: AHI = apnea–hypopnea index, BMI = body mass index, CVD = cardiovascular disease, ESS = Epworth Sleepiness Scale, FPG = fasting plasma glucose, HDL‐C = high‐density lipoprotein cholesterol, LDL‐C = low‐density lipoprotein cholesterol, LSaO2 = lowest pulse oxygen saturation, METS‐IR = metabolic score of insulin resistance, MSaO2 = mean pulse oxygen saturation, ODI = oxygen desaturation index, RH = resistant hypertension, TC = total cholesterol, TG = triglyceride, TST = total sleep time, T90 = percentage of total time with oxygen saturation level < 90%.

^a^ χ^2^ test.

^b^ Kruskal–Wallis test.

^c^ ANOVA.

Multivariable logistic regression analyses were performed using clinically relevant variables. Model 1 was unadjusted. Model 2 was adjusted for age, sex, alcohol consumption, cerebral infarction, diabetes, and CVD. Model 3 was further adjusted for sleep‐respiratory parameters and lipid profiles to control for potential confounding factors.

For Hypertension: In the unadjusted Model 1, the risks for hypertension in the M2, M3, and M4 groups were significantly elevated compared to the reference group, with ORs of 2.413 (95% CI: 1.173–4.965, *p* = 0.017), 2.221 (95% CI: 1.056–4.669, *p* = 0.035), and 2.239 (95% CI: 1.042–4.811, *p* = 0.039), respectively. A significant linear dose–response relationship was observed (*p‐trend* = 0.027). In Model 2, adjusted for age, sex, and comorbidities, the risk remained significant only for the M2 group (OR = 2.260, 95% CI: 1.070–4.774, *p* = 0.033), while the associations for the M3 and M4 groups became nonsignificant. This pattern persisted in the fully adjusted Model 3 (further adjusted for sleep‐respiratory parameters and lipid profiles), where a significant association was maintained solely for the M2 group (OR = 2.160, 95% CI: 1.015–4.596, *p* = 0.046). The associations for M3 and M4 were attenuated, and the linear trend in Model 3 was not statistically significant (*p‐trend* = 0.254) (Table 7).

**TABLE 7:** Multivariate logistic regression analysis of METS‐IR level on OSA complicated with hypertension/RH in non‐severe OSA group.

METS‐IR/hypertension, RH	Hypertension						RH					
	Model 1		Model 2		Model 3		Model 1		Model 2		Model 3	
	OR (95% CI)	*p*	OR (95% CI)	*p*	OR (95% CI)	*p*	OR (95% CI)	*p*	OR (95% CI)	*p*	OR (95% CI)	*p*
M1	—	—	—	—	—	—	—	—	—	—	—	—
M2	2.413 (1.173, 4.965)	0.017	2.260 (1.070, 4.774)	0.033	2.160 (1.015, 4.596)	0.046	2.026 (0.721, 5.691)	0.180	2.380 (0.822, 6.891)	0.110	2.422 (0.816, 7.190)	0.111
M3	2.221 (1.056, 4.669)	0.035	1.990 (0.872, 4.543)	0.102	1.882 (0.818, 4.330)	0.137	2.406 (0.854, 6.783)	0.097	2.406 (0.788, 7.346)	0.123	1.933 (0.611, 6.112)	0.262
M4	2.239 (1.042, 4.811)	0.039	1.610 (0.621, 4.177)	0.328	1.448 (0.538, 3.896)	0.464	6.210 (2.388, 16.149)	< 0.001	4.978 (1.537, 16.128)	0.007	2.948 (0.853, 10.184)	0.087
*p‐trend*		0.027		0.186		0.254		< 0.001		0.011		0.132

*Note:* Model 1 was unadjusted; Model 2 was adjusted for age, sex, alcohol consumption, cerebral infarction, diabetes, and CVD; and Model 3 was further adjusted for sleep‐related parameters, including T90, LSaO2, MSaO2, ODI, and TC, based on Model 2.

Abbreviation: RH = resistant hypertension.

For RH: In the unadjusted Model 1, a robust and significant association was observed specifically in the highest quartile (M4), with an OR of 6.210 (95% CI: 2.388– *p* < 0.001), while no significant differences were found in the M2 and M3 groups. The trend test demonstrated a highly significant linear relationship (*p‐trend* < 0.001). In Model 2, the M4 group retained a significant positive association with RH (OR = 4.978, 95% CI: 1.537– *p* = 0.007), and the linear trend remained significant (*p‐trend* = 0.011). However, in the fully adjusted Model 3, the association in the M4 group was attenuated and lost statistical significance (OR = 2.948, 95% CI: 0.853–10.184, *p* = 0.087). Similarly, the linear dose–response relationship was no longer significant (*p‐trend* = 0.132) after controlling for sleep parameters and lipid profiles. Detailed results are presented in Table 7.

### 3.6 Discriminative Superiority and Statistical Power

We further evaluated whether the composite METS‐IR index outperformed simple anthropometric (BMI) or lipid (TG/HDL) indices.

**FIGURE 2:**
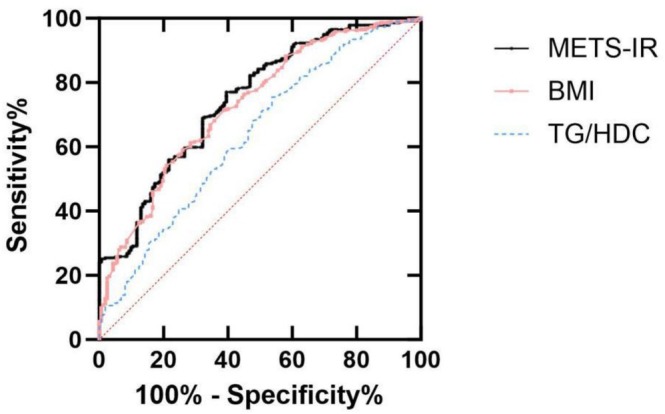
Discriminative value of METS‐IR, BMI, and TG/HDL for hypertension.

For hypertension: The AUC for METS‐IR was 0.745 (95% CI: 0.703–0.788, *p* < 0.05), which was significantly greater than the AUC for BMI, which was 0.729 (95% CI: 0.685–0.733, *p* < 0.05), and TG/HDL, which was 0.636 (95% CI: 0.568–0.686, *p* < 0.05) (Figure 2).

**FIGURE 3:**
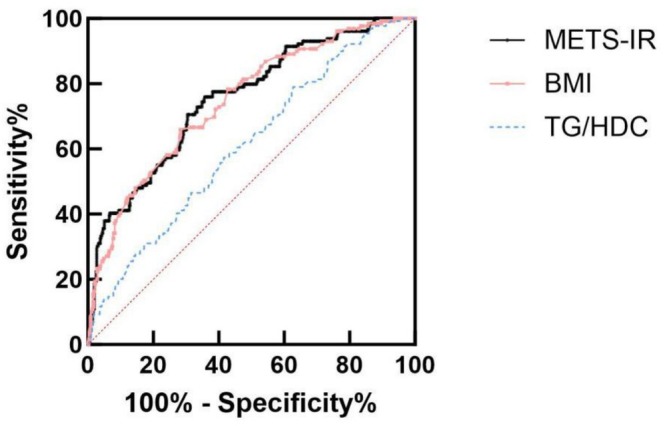
Discriminative value of METS‐IR, BMI, and TG/HDL for RH.

For RH: METS‐IR also showed the strongest discriminative power with an AUC of 0.754 (95% CI: 0.707–0.801, *p* < 0.05), surpassing both BMI as 0.746 (95% CI: 0.699–0.793, *p* < 0.05) and TG/HDL as 0.610 (95% CI: 0.557–0.663, *p* < 0.05) (Figure 3).

These findings indicate that while both adiposity and lipid dysregulation contribute to risk, their integration within the METS‐IR index provides a more comprehensive and accurate discriminative tool for hypertensive states in OSA patients.

Finally, post hoc power analyses based on the comparison between extreme quartiles (M4 vs. M1) were conducted. The analyses demonstrated robust statistical power for both the total cohort (power > 0.99 for hypertension; power = 0.98 for RH) and the severe OSA subgroup (power > 0.99; power = 0.98). Conversely, the non‐severe OSA subgroup was underpowered (power = 0.15 and 0.46 for hypertension and RH, respectively). This limited statistical power likely explains the lack of significant associations in this milder OSA stratum.

## 4 Discussion

While the relationship between sleep apnea, insulin resistance, and hypertension has been extensively studied using classic markers like Homeostasis Model Assessment of Insulin Resistance (HOMA‐IR), this study specifically examines the strength of the association of the composite METS‐IR index with both hypertension and RH across different OSA severities. Our findings demonstrate a significant linear dose–response relationship between elevated METS‐IR levels and increased risks of hypertension and RH, which was particularly robust in patients with severe OSA. In contrast, this association was attenuated and lacked a significant linear trend in the non‐severe OSA group. These results suggest that the association between METS‐IR and hypertension is modified by OSA severity. While this modification may be driven by a compounding effect of severe hypoxic burden on insulin resistance, we cannot rule out that the weaker association observed in non‐severe patients is a reflection of lower baseline metabolic burden and a narrower range of metabolic derangement in this subgroup, rather than distinct underlying pathophysiological mechanisms.

A strong linear dose–response relationship became apparent in the severe OSA subgroup whereby the relative increases in the quartiles of METS‐IR were correlated with an increase in risks of hypertension and RH. This finding is consistent with the results of previous studies, which have found IR to play a central role in this group of patients [15]. Chronic intermittent hypoxia is a typical feature of severe OSA that leads to the activity of the sympathetic nervous system, oxidative stress, and release of pro‐inflammatory cytokines. These pathophysiologic mechanisms enhance IR that subsequently perpetuates hypertension [16]. Also, IR contributes to dysregulation of blood pressure by causing renin‐angiotensin‐aldosterone system (RAAS) activation and encouragement of sodium and water retention, which further increases the likelihood of hypertension and RH [17].

The association in the non‐severe OSA subgroup was significantly diluted, and it did not exhibit a statistically significant linear association. Despite the fact that the risk in patients was highest in higher levels of METS‐IR, this tendency was not significant. Several hypotheses may explain the observed divergence in association strengths between the two subgroups: (1) similar, less significant hypoxic load: The non‐severe form of intermittent hypoxia probably results in less sympathetic activation, oxidative stress, and inflammation and attenuating its effect on hypertension and RH by IR. Murphy et al. have found this hypothesis to be supported as they have described that intermittent hypoxia spreads IR during adipose‐tissue inflammation that more serious hypoxia worsens the inflammatory cascade [15]; Giampá et al. have also noted that milder hypoxia in non‐severe OSA lessens the metabolic and cardiovascular burden [18]. Sforza and Roche have found a less severe induction of IR and its subsequent [19] (2) subclinical metabolic dysfunction: The metabolic imbalances in patients with non‐severe OSA might be less severe, and therefore, the IR is not robust enough to initiate hypertension or RH independently. Rühle et al. proved that severe IR is less relevant to the pathogenesis of hypertension [20], and Yeghiazarians et al. have shown that non‐severe OSA predisposes mild cases of cardiovascular malfunctions including hypertension and RH [21]. It could be possible that (3) confounding influences within cohorts with non‐severe OSA may conceal the actual association between OSA severity and IR, and blood pressure outcomes, due to the higher frequency of confounders, including obesity and diabetes mellitus [22].

Having established these varying associations across OSA severities, our study also addressed the critical methodological question of whether METS‐IR offers incremental value beyond traditional metrics like BMI. Through ROC curve analysis, we formally demonstrated that METS‐IR is a more precise marker for both hypertension and RH. This superiority stems from its ability to integrate adiposity with lipid and glucose dysregulation, capturing the multifaceted nature of insulin resistance that BMI alone overlooks.

Furthermore, multiple studies have demonstrated that METS‐IR exhibits superior discriminative capacity compared to other established metabolic indices, such as the HOMA‐IR [23]. This indicates that METS‐IR possesses robust validity and practical operability in the context of metabolic diseases, highlighting its considerable potential for broader application in the identification of individuals at high cardiovascular risk. In a retrospective study based on a health‐examination population, Li et al. [24]. compared the discriminative capacity of the TyG index and METS‐IR for identifying concurrent non‐alcoholic fatty liver disease (NAFLD) in patients with OSA. Their results showed that while both IR indices were significantly associated with NAFLD risk, the discriminative performance of METS‐IR was slightly superior to that of the TyG index (AUC: 0.778 vs. 0.775), with higher METS‐IR levels indicating a significantly increased risk of NAFLD in a dose–response manner. These findings suggest that by integrating BMI, glucose, and lipid profiles, METS‐IR provides a more comprehensive assessment of metabolic burden, thereby facilitating the identification of OSA patients at high risk for NAFLD. Similarly, our study found that OSA patients in the high METS‐IR group exhibited more severe metabolic abnormalities—characterized by elevated BMI, FPG, TG, TC, and HDL‐C. Furthermore, this group demonstrated worsened sleep respiratory parameters (i.e., increased AHI, ODI, and T90%, with decreased LSaO_2_ and MSaO_2_) and an increased burden of cardiovascular disease. From a different perspective, our results corroborate Li's viewpoint: In the OSA population, METS‐IR not only reflects the degree of insulin resistance but may also unmask the synergistic interaction between OSA and metabolic burden, thereby providing a crucial basis for clinical risk stratification and early intervention.

Moreover, the high statistical power (Power > 0.80) obtained in our post hoc analysis reinforces the validity of these overarching findings. It suggests that the observed increased risks across METS‐IR quartiles are not due to chance but represent a robust biological relationship. While some intermediate quartiles in the non‐severe subgroups showed nonsignificant trends, our power analysis confirms that the overall study was sufficiently powered to detect the primary metabolic impact of METS‐IR on blood pressure regulation.

This study demonstrates that METS‐IR is independently associated with hypertension and RH in this cohort of patients with OSA. The robust dose–response correlation in patients with severe OSA highlights the potential of METS‐IR as a simple composite metabolic parameter to aid in clinical risk stratification. Clinicians are therefore advised to early look into intensive metabolic interventions such as weight reduction programs and interventions geared toward the enhancement of insulin sensitivity to patients with severe OSA and a high level of METS‐IR that will complement the usual OSA management to maximize the blood pressure in the patients. In contrast, the utility of METS‐IR for risk assessment alone appears limited in the context of people who have non‐severe OSA; in this subgroup, a broader evaluation that would entail BMI, AHI, and other metabolic values is a prerequisite to having the right risk evaluation.

Despite providing valuable insights, this study has several limitations. First, its single‐center, cross‐sectional design precludes establishing causal relationships. To better validate METS‐IR as an independent marker of future hypertension, future prospective, multi‐center cohort studies are necessary. Second, this study did not compare METS‐IR with other established measures of insulin resistance, such as HOMA‐IR, preventing the determination of its relative superiority or inferiority in predicting hypertension and RH. Additionally, because the METS‐IR quartiles were defined based on the entire study population, the distribution of participants across quartiles within the severe OSA (M1: 59, M4: 86) and non‐severe OSA (M1: 111, M4: 84) subgroups was uneven. This uneven group distribution could affect the precision of the estimated odds ratios and should be considered an analytical limitation. Finally, METS‐IR is based on static biochemical parameters, which do not capture dynamic metabolic fluctuations, such as postprandial glucose variability. To validate METS‐IR as a reliable risk indicator in clinical practice, future multi‐center prospective cohorts are needed to assess its dynamic changes and long‐term clinical associations. Furthermore, integrating dynamic metabolic assessments, such as continuous glucose monitoring, with molecular biomarkers of inflammation and oxidative stress could provide deeper insights into the mechanistic pathways linking METS‐IR, insulin resistance, and cardiovascular outcomes in OSA.

## Conclusions

In conclusion, elevated METS‐IR is independently associated with an increased prevalence of hypertension and resistant hypertension in patients with severe OSA, exhibiting a clear dose–response pattern. However, its utility for risk assessment appears limited in non‐severe OSA, where it should be interpreted cautiously alongside traditional metabolic parameters. These findings support the potential of METS‐IR as a simple, cost‐effective tool for early cardiovascular risk stratification in severe OSA phenotypes.

## Author Contributions

Jinwei Xin designed this study, conducted data analysis, and drafted the manuscript. Taofeng Zhu, as the corresponding author, supervised the research process, critically reviewed, and revised the manuscript. Rongxiu Xie, Wenxu Zhou, Jiaying Li, and Chen Zhou assisted with data collection, statistical analysis, and manuscript preparation. All authors read and approved the final version of the manuscript.

## Funding

This work was supported by the National Natural Science Foundation of China (Grant No. 81802102) and the Wuxi Health Commission's Middle‐Aged and Young Talent Project (Grant No. BJ2023105). The funding bodies had no role in study design, data collection, analysis, interpretation, or manuscript writing.

## Ethics Statement

All procedures performed in studies involving human participants were conducted in accordance with the ethical standards of the institutional and/or national research committee, specifically the Medical Ethics Committee of the Affiliated Yixing Hospital of Jiangsu University (Approval No. 2023‐040), and with the 1964 Helsinki Declaration and its later amendments or comparable ethical standards. This study was a retrospective analysis of anonymized clinical data; therefore, formal written consent was not required.

## Consent

The authors have nothing to report.

## Conflicts of Interest

The authors declare no conflicts of interest.

## Data Availability

The datasets used and analyzed in this study are available from the corresponding author upon reasonable request.
